# Internal living environment and respiratory disease in children: findings from the *Growing Up in New Zealand* longitudinal child cohort study

**DOI:** 10.1186/s12940-016-0207-z

**Published:** 2016-12-08

**Authors:** Sandar Tin Tin, Alistair Woodward, Rajneeta Saraf, Sarah Berry, Polly Atatoa Carr, Susan M. B. Morton, Cameron C. Grant

**Affiliations:** 1 Section of Epidemiology and Biostatistics, School of Population Health, University of Auckland, Private Bag 92019, Auckland, 1142 New Zealand; 2 Centre for Longitudinal Research - He Ara ki Mua and Growing Up in New Zealand, University of Auckland, Private Bag 92019, Auckland, 1142 New Zealand; 3 National Institute of Demographic and Economic Analysis, University of Waikato and Waikato District Health Board, Hamilton, New Zealand; 4 Department of Paediatrics: Child and Youth Health, University of Auckland, Private Bag 92019, Auckland, 1142 New Zealand; 5 Starship Children’s Hospital, Auckland District Health Board, Auckland, New Zealand

**Keywords:** Indoor air pollution, Housing, Heating, Crowding, Smoking, Respiratory tract infections, Hospitalisation, Preschool children, Cohort study

## Abstract

**Background:**

The incidence of early childhood acute respiratory infections (ARIs) has been associated with aspects of the indoor environment. In recent years, public awareness about some of these environmental issues has increased, including new laws and subsequent changes in occupant behaviours. This New Zealand study investigated current exposures to specific risk factors in the home during the first five years of life and provided updated evidence on the links between the home environment and childhood ARI hospitalisation.

**Methods:**

Pregnant women (*n* = 6822) were recruited in 2009 and 2010, and their 6853 children created a child cohort that was representative of New Zealand births from 2007-10. Longitudinal data were collected through face-to-face interviews and linkage to routinely collected national datasets. Incidence rates with Poisson distribution confidence intervals were computed and Cox regression modelling for repeated events was performed.

**Results:**

Living in a rented dwelling (48%), household crowding (22%) or dampness (20%); and, in the child’s room, heavy condensation (20%) or mould or mildew on walls or ceilings (13%) were prevalent. In 14% of the households, the mother smoked cigarettes and in 30%, other household members smoked. Electric heaters were commonly used, followed by wood, flued gas and unflued portable gas heaters. The incidence of ARI hospitalisation before age five years was 33/1000 person-years. The risk of ARI hospitalisation was higher for children living in households where there was a gas heater in the child’s bedroom: hazard ratio for flued gas heater 1.69 (95% CI: 1.21-2.36); and for unflued gas heater 1.68 (95% CI: 1.12-2.53); and where a gas heater was the sole type of household heating (hazard ratio: 1.64 (95% CI: 1.29-2.09)). The risk was reduced in households that used electric heaters (Hazard ratio: 0.74 (95% CI: 0.61-0.89)) or wood burners (hazard ratio: 0.79 (95% CI: 0.66-0.93)) as a form of household heating. The associations with other risk factors were not significant.

**Conclusions:**

The risk of early childhood ARI hospitalisation is increased by gas heater usage, specifically in the child’s bedroom. Use of non-gas forms of heating may reduce the risk of early childhood ARI hospitalisation.

**Electronic supplementary material:**

The online version of this article (doi:10.1186/s12940-016-0207-z) contains supplementary material, which is available to authorized users.

## Background

Acute respiratory infections (ARIs) occur frequently in early childhood, and account for many primary care visits and hospital admissions [1]. Compared to other developed countries such as England and the United States, New Zealand (NZ) has relatively high childhood hospital admission rates for ARIs [2–5]. Additionally in NZ, childhood ARI hospitalisation rates vary significantly between population subgroups, with the highest burden observed in children of Māori (NZ’s indigenous population) or Pacific ethnicity and those living in socially deprived areas [2, 6, 7].

As young children spend most of their time in the home, the indoor environment has the potential to play an important role in their susceptibility to illnesses caused by respiratory infections. Specific aspects of the indoor living environment such as household dampness and mould, and residential crowding have been shown to increase the risk of ARIs and hospital admission with ARIs in children [8–11]. Exposures to indoor air pollutants produced by heating or cooking or from cigarette smoking have also been associated with an increased risk of ARI in early childhood [12–14].

In recent years, public awareness about some of these environmental issues has increased, responding to new laws such as Smoke-free Environments Act and subsequent changes in behaviour. For example, exposure to second hand smoke has greatly decreased in the UK, the US and also in NZ [15–18]. However, there have been few legislative changes affecting other environmental exposures in the home, particularly heating, in NZ.

Our aim was to investigate, in a birth cohort representative of recent New Zealand births, the frequency and pattern of exposure to specific home environmental risk factors (household tenure, crowding, household smoking, forms of heating, dampness of the house, heavy condensation and mould or mildew in the child’s bedroom) and to provide updated evidence of the impact of these exposures on the risk of hospital admission with ARIs during the first five years of life.

## Methods

### Design, setting and participants

We completed our study within *Growing Up in New Zealand,* NZ’s contemporary child cohort study. Essential design features of this study were recruitment during pregnancy, inclusion of partners and enrolment of an ethnically and socioeconomically diverse sample that would be generalizable to all national births [19]. To be eligible pregnant women had to be resident within a geographical region defined by three contiguous District Health Board (DHB) regions in the northern part of the country (Auckland, Counties-Manukau and Waikato), and to have an estimated delivery date between 25 April 2009 and 25 March 2010. The Auckland and Counties-Manukau DHB regions experience a subtropical climate with mean annual temperatures between 14 °C and 16 °C [20]. In the Waikato DHB region, the landscape setting varies widely and so does the climate with mean annual temperatures ranging from less than 8 °C at higher elevations to 14 °C in the low-lying areas [21]. The 6853 children enrolled represent 11% of national births during the recruitment period, and the characteristics of the study cohort closely align with those of recent national births [22]. Ethical approval for the study was granted by the NZ Ministry of Health Northern Y Regional Ethics committee and written informed consent provided by all participants for study participation and data linkage.

**Fig. 1:**
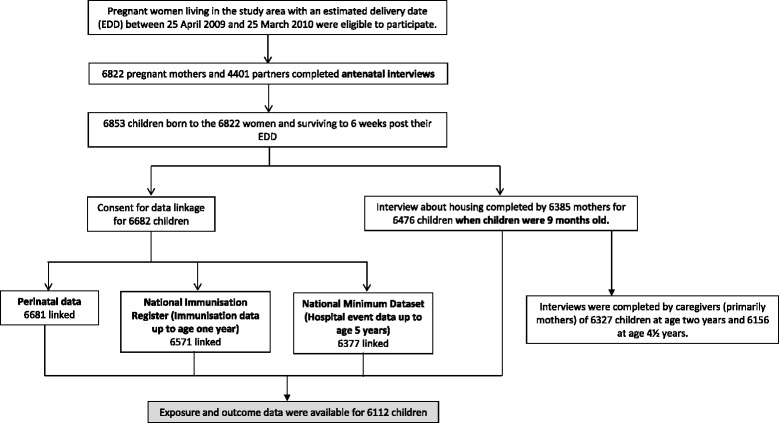
Antenatal recruitment of parents of cohort children and data collection about home environmental risk factors, hospital admissions with acute respiratory infections and confounding variables

Data were collected through computer assisted face-to-face personal interviews (CAPI), telephone interviews and linkage to administrative datasets. Linked datasets included those in which are stored perinatal health records, the scheduled childhood immunisations [23] and hospital events. In NZ all acute paediatric hospital admissions are to public hospitals and these healthcare events are recorded in the NZ Ministry of Health National Minimum Dataset [24]. Figure 1 summarises study participant recruitment and retention and completeness of data collection and linkage.

### Measurements

Computer-assisted-personal-interviews (CAPIs) were completed with each pregnant woman at enrolment and then when her child was nine months, two and 4½ years old. At these interviews information was collected across six interconnected research domains: societal context and neighbourhoods, culture and identity, family and whānau (extended family), education, psychological and cognitive development, and health and well-being. The analysis reported here used data collected from the CAPIs administered antenatally (variables describing maternal demographics, health status, use of supplements, smoking and pregnancy history); and when the cohort children were nine months old (variables describing child health, feeding practices, time spent outdoors and internal household environmental measures).

The internal household environmental factors were measured when the children were nine months old and were mostly reported by the biological mother (99.6%). These include: household tenure, crowding, household smoking, forms of heating used in the house and specifically in the room where the cohort child slept at night (electricity, flued gas heater, unflued gas heater, wood, coal and others), dampness of the house, heavy condensation and mould or mildew in the cohort child’s bedroom. Household crowding was measured as a ratio of household occupants to bedrooms, with a ratio of ≥2 used to define a higher level of crowding.

### Acute respiratory infections

ARI hospital admissions were determined through linkage to the national database of hospital events stored by the Ministry of Health in the National Minimum Dataset covering the time period from the cohort child’s date of birth until their fifth birthday. The dataset contains information about inpatient and day patient hospital events from all public hospitals and over 90% of private hospitals [24]. For each event discharge diagnoses are documented using ICD-10-AM codes.

### Statistical Analyses

We restricted our analysis to the data about the 6112 children whose mothers completed the CAPI that occurred when their infant was 9 months old and for whom linkage was established with hospital event data. Missing values were computed using multiple imputation with ten complete datasets created by the Markov chain Monte Carlo method, incorporating maternal, child and all household factors listed in Table 1 and in Additional file 1: Table S2 and S3 and ARI hospital admissions. The percentage of missing data was highest for pre-pregnancy BMI (11.7%), maternal use of supplements (9.5%), maternal smoking (9.5%), household tenure (7.7%) and child immunisation (10.2%), and was at most 1.2% for other variables.

**Table 1:** Characteristics of the internal living environment as described at age nine months for 6112 cohort children

Internal living environment	N (%)
Housing tenure (*N* = 5640)	
Owner occupancy	2916 (52)
Tenancy	2724 (48)
Crowding index^a^ (*N* = 6111)	
<1 (low)	336 (6)
1- < 2 (medium)	4430 (72)
2+ (high)	1345 (22)
Maternal smoking (*N* = 6110)	
Yes	870 (14)
No	5240 (86)
Smoking by others in the household (*N* = 6109)	
Yes	1835 (30)
No	4274 (70)
Heating the house (*N* = 6108)	
Yes	5449 (89)
No	659 (11)
Form/s of heating used in the house^b^ (*N* = 5447)	
Electric heater	4854 (89)
Flued gas heater	769 (14)
Unflued portable gas heater	671 (12)
Wood burner	1498 (28)
Coal burner	96 (2)
Others	72 (1)
Form/s of heating used in the room where child sleeps at night^b^ (*N* = 5297)	
No heating	763 (14)
Electric heater	4337 (82)
Flued gas heater	138 (3)
Unflued portable gas heater	74 (1)
Wood burner	243 (5)
Coal burner	16 (0)
Others	39 (1)
Dampness of the house (*N* = 6070)	
Never or hardly ever	3053 (50)
Not very often	1772 (29)
Quite often	908 (15)
Always or almost always	337 (6)
Heavy condensation in the room where baby sleeps at night (*N* = 6084)	
Never or hardly ever	3109 (51)
Not very often	1654 (27)
Quite often	951 (16)
Always or almost always	370 (6)
Mould or mildew in the walls or ceilings in the room where child sleeps at night in the past two weeks (*N* = 6101)	
Yes	769 (13)
No	5332 (87)

^a^Household crowding was measured as a ratio of household occupants to bedrooms

^b^Multiple responses allowed

An ARI hospital admission was defined as a hospital event that involved at least one overnight hospital stay and with one of the following ICD principal discharge diagnosis codes: A15, A16, A19, A36, A37, A71, B26, B30, H00, H01, H04, H10, H60, H65, H66, H67, H70-H75, H83, J00-J06, J09-J18, J20-J22, J30-J39, J40-J47, J86, J90-J93, J95, J96, J98 and J99 (see details in Additional file 1: Table S1). Readmissions for the same illness (within 3 days of discharge from the index hospitalisation) were excluded (*n* = 97). Hospital events for which the hospital admission and discharge date were the same were excluded (*n* = 963).

As some children had more than one ARI hospital admission before their 5th birthday, incidence rates of repeated events were calculated using the person-years approach. Confidence intervals were based on the Poisson distribution. The participants were censored on their fifth birthday or date of death.

Cox proportional hazards regression modelling for repeated events was performed using a counting process approach and indoor environmental factors influencing the likelihood of experiencing ARI episodes were identified. For the pregnancies that had resulted in a multiple birth, only the first child born was included in the analysis, resulting in exclusion of 93 children.

Three separate models were created with stepwise adjustment for (i) antenatal maternal factors (demographics, health status, use of supplements, smoking and pregnancy history); then for (ii) child factors (gender, gestation, birth-weight, season of birth, health status, feeding practices, sun exposure and immunisation), and lastly for (iii) indoor environmental factors. Associations were described using hazards ratios (HR) and 95% confidence intervals (CI). Some households used more than one form of heating and as using a gas heater was found to be significantly associated with hospitalisation for an ARI, the hazard associated with using a gas heater solely versus in combination with other forms of heating was also investigated.

Three separate sensitivity analyses were undertaken by restricting the study sample to those who did not move home between the antenatal and 2 year interviews, by restricting the follow-up period to age 2 years and by restricting the outcome events to ARIs that had occurred in the winter and spring (June-November), when heating was most likely to be required, respectively. SAS (release 9.4, SAS Institute Inc., Cary, North Carolina) was used for all analyses.

## Results

The characteristics of the mothers of the cohort children who were included compared with those excluded from this analysis (whose mothers did not complete the CAPI that was administered when their infant was 9 months old or for whom linkage was not established with hospital event data) are presented in Additional file 1: Table S2. In comparison with mothers of the cohort children who were excluded from the analysis (*n* = 648) a larger proportion of the mothers of the cohort children who were included in the analysis (*n* = 6112) were of European ethnicity (55% vs. 39%, *p* < 0.001); had a tertiary education (70% vs. 61%, *p* < 0.001); and a smaller proportion resided in the most deprived quintile of NZ households (26% vs. 38%, *p* < 0.001). The mothers of the cohort children included in this analysis had a higher self-rated pre-pregnancy health rating (*p* = 0.02). A smaller proportion of them had smoked cigarettes during pregnancy (9% vs. 13%). A larger proportion had used folic acid supplements (40% vs. 29%, *p* < 0.001 prior to pregnancy; 81% vs. 66%, *p* < 0.001 in the first three months of pregnancy and 56% vs. 48%, *p* = 0.002 since the first three months of pregnancy) or had used vitamin and mineral supplements (31% vs. 22%, *p* < 0.001 prior to pregnancy; 51% vs. 39%, *p* < 0.001 in the first three months of pregnancy and 53% vs. 46%, *p* = 0.002 since the first three months of pregnancy). For a larger proportion this was a planned pregnancy (61% vs. 52%, *p* < 0.001).

The characteristics of the cohort children who were included compared with those excluded from this analysis are presented in Additional file 1: Table S3. In comparison with the cohort children who were excluded from the analysis (*n* = 648) the mean birth weight of the cohort children who were included in the analysis (*n* = 6112) was slightly lower (3496.8 g vs. 3499.1 g; *p* = 0.02) but there were no significant differences by gender, gestation and season of birth. Comparisons were not made for other child characteristics as a large proportion of the excluded group had missing values.

Almost half of the mothers reported living in a rented dwelling and 22% lived in a household with a higher level of crowding (Table 1). At age 9 months approximately 1-in-7 of the children (14%) had a mother who currently smoked cigarettes and 30% were living with other household members who smoked.

Some form of heating was reported to be used in 89% of the households, with the most commonly reported form of heating being electric heater (80%) followed by wood burners (5%), flued gas heater (3%) and unflued portable gas heater (1%).

For approximately 20% of the households, dampness in the house or heavy condensation in the room where the child slept at night were reported to be present quite often, almost always or always. For approximately 13% of the households mould or mildew was reported to be present in the walls or ceilings of the room where the child slept.

A total of 708 children experienced 1017 ARI hospital admissions in the first five years of life, corresponding to 33 admissions per 1000 person-years (95% CI: 31.25-35.37) (Table 2).

**Table 2:** Number of cohort children admitted to hospital for an acute respiratory infection during the first five years of life

No. of acute respiratory infection events requiring hospital admission	N (%)
1	553 (9)
2	91 (2)
3	28 (1)
4	18 (0)
5+	18 (0)
Total number of children	708 (12)
Total number of events	1017
Total number of person-years	30576.73
Incidence per 1000 person-years (95% CI)	33.26 (31.25-35.37)

In the multivariable analysis that adjusted for maternal, child and all household factors (listed in Additional file 1: Table S2 and S3 and in Table 1), using a flued (HR = 1.69, 95% CI 1.21-2.36) or unflued gas (HR = 1.68, 95% CI 1.12-2.53) heater in the room where the child slept at night was associated with an increased risk of being hospitalised with an ARI during the first five years (Table 3). Using electricity or wood for household heating and reporting mould or mildew in the child’s room were associated with a reduced risk of ARI hospitalisation.

**Table 3:** Associations between internal living environment and hospital admission for an acute respiratory infection during the first five years of life

Variable	Incidence per 1000 person-years	Crude HR (95% CI)	Adjusted HR1^a^ (95% CI)	Adjusted HR2^b^ (95% CI)	Adjusted HR3 (95% CI)
Housing tenure					
Owner occupancy	27.73 (25.19-30.46)	1.00	1.00	1.00	1.00
Tenancy	39.11 (36.00-42.43)	1.41 (1.22-1.63)	1.02 (0.88-1.18)	1.02 (0.88-1.18)	1.00 (0.87-1.16)^c^
Crowding index					
<1-2 (low and medium)	29.19 (27.06-31.44)	1.00	1.00	1.00	1.00
2+ (high)	47.68 (42.61-53.19)	1.63 (1.42-1.88)	1.11 (0.95-1.29)	1.07 (0.91-1.25)	1.07 (0.91-1.26)^c^
Maternal cigarette smoking					
Yes	55.23 (48.47-62.66)	1.87 (1.60-2.18)	1.14 (0.90-1.45)	1.06 (0.83-1.36)	1.10 (0.84-1.44)^c^
No	29.61 (27.57-31.77)	1.00	1.00	1.00	1.00
Cigarette smoking by others in the household					
Yes	44.58 (40.36-49.11)	1.57 (1.37-1.80)	0.99 (0.85-1.16)	0.97 (0.83-1.14)	0.94 (0.79-1.11)^c^
No	28.40 (26.19-30.75)	1.00	1.00	1.00	1.00
Heating the house					
Yes	31.41 (29.34-33.58)	0.65 (0.54-0.78)	0.99 (0.82-1.19)	0.87 (0.72-1.06)	0.87 (0.72-1.05)^c^
No	48.58 (41.35-56.71)	1.00	1.00	1.00	1.00
Form/s of heating used in the house^d^ (*N* = 5298)					
Electric heater					
Yes	28.97 (26.87-31.19)	0.56 (0.47-0.68)	0.70 (0.58-0.83)	0.74 (0.61-0.89)	0.74 (0.61-0.89)^e^
No	51.33 (43.52-60.15)	1.00	1.00	1.00	1.00
Flued gas heater					
Yes	31.95 (26.55-38.12)	1.02 (0.83-1.25)	1.12 (0.92-1.37)	1.07 (0.88-1.31)	1.08 (0.89-1.32)^e^
No	31.32 (29.09-33.67)	1.00	1.00	1.00	1.00
Unflued portable gas heater					
Yes	31.91 (26.15-38.55)	1.02 (0.82-1.27)	0.89 (0.73-1.10)	0.92 (0.75-1.13)	0.90 (0.73-1.11)^e^
No	31.34 (29.13-33.66)	1.00	1.00	1.00	1.00
Wood burner					
Yes	26.70 (23.13-30.67)	0.80 (0.68-0.95)	0.79 (0.67-0.94)	0.78 (0.66-0.93)	0.79 (0.66-0.93)^e^
No	33.19 (30.70-35.83)	1.00	1.00	1.00	1.00
Form/s of heating used in the room where child sleeps at night^d^ (*N* = 5298)					
No heating					
Yes	32.33 (26.89-38.56)	1.03 (0.84-1.27)	0.76 (0.63-0.93)	0.82 (0.67-0.99)	0.82 (0.67-1.00)^e^
No	31.26 (29.03-33.60)	1.00	1.00	1.00	1.00
Electric heater					
Yes	29.27 (27.04-31.64)	0.74 (0.62-0.87)	0.95 (0.81-1.12)	0.94 (0.80-1.11)	0.94 (0.80-1.10)^e^
No	39.74 (34.68-45.33)	1.00	1.00	1.00	1.00
Flued gas heater					
Yes	60.79 (43.81-82.17)	1.98 (1.42-2.77)	1.96 (1.43-2.68)	1.69 (1.21-2.36)	1.69 (1.21-2.36)^e^
No	30.64 (28.58-32.82)	1.00	1.00	1.00	1.00
Unflued portable gas heater					
Yes	70.04 (45.75-102.63)	2.27 (1.49-3.45)	1.78 (1.20-2.64)	1.68 (1.12-2.36)	1.68 (1.12-2.53)^e^
No	30.87 (28.81-33.05)	1.00	1.00	1.00	1.00
Wood burner					
Yes	32.08 (22.81-43.86)	1.02 (0.72-1.44)	0.93 (0.67-1.29)	1.01 (0.73-1.41)	1.02 (0.73-1.42)^e^
No	31.38 (29.26-33.60)	1.00	1.00	1.00	1.00
Dampness of the house					
Never or hardly ever	27.86 (25.15-30.77)	1.00	1.00	1.00	1.00
Not very often	30.79 (27.09-34.85)	1.07 (0.92-1.25)	0.93 (0.79-1.08)	0.95 (0.82-1.11)	0.96 (0.83-1.13)^c^
Quite often	38.62 (32.71-45.29)	1.47 (1.23-1.75)	1.13 (0.95-1.34)	1.08 (0.91-1.29)	1.13 (0.94-1.36)^c^
Always or almost always	51.30 (39.81-65.07)	1.81 (1.43-2.30)	1.13 (0.89-1.43)	1.07 (0.84-1.37)	1.15 (0.89-1.50)^c^
Heavy condensation in the room where child sleeps at night					
Never or hardly ever	29.90 (27.12-32.89)	1.00	1.00	1.00	1.00
Not very often	29.61 (25.84-33.76)	1.05 (0.90-1.23)	0.95 (0.81-1.11)	1.00 (0.86-1.17)	1.01 (0.86-1.17)^f^
Quite often	35.63 (30.12-41.87)	1.29 (1.08-1.54)	1.08 (0.90-1.28)	1.02 (0.86-1.22)	1.05 (0.88-1.27)^f^
Always or almost always	43.04 (33.15-54.95)	1.47 (1.15-1.86)	0.97 (0.76-1.23)	0.94 (0.74-1.20)	1.00 (0.77-1.31)^f^
Mould or mildew in the walls or ceilings in the room where child sleeps at night in the past two weeks					
Yes	33.88 (27.66-41.08)	1.24 (1.03-1.49)	0.92 (0.77-1.10)	0.88 (0.73-1.41)	0.81 (0.67-0.99)^c^
No	31.10 (28.92-33.40)	1.00	1.00	1.00	1.00

^a^Adjusted for all maternal factors (age, ethnicity, education, area of residence, neighbourhood deprivation, pre-pregnancy BMI, pre-pregnancy self-rated health, history of asthma, use of supplements, maternal smoking, parity and pregnancy planning)

^b^Adjusted for all maternal factors as above and all child factors (gender, gestation, birth-weight, season of birth, proxy-rated health at 9 months, health or developmental problems, feeding practices, time spent outdoors and child immunisation)

^c^Adjusted for all maternal and child factors as above and each other

^d^Restricted to those who reported heating the house

^e^Adjusted for all maternal and child factors as above and environmental factors (household tenure, crowding, household smoking, dampness of the house and mould or mildew in the child’s bedroom)

^f^Adjusted for all maternal and child factors as above and environmental factors (household tenure, crowding, household smoking, heating the hose and mould or mildew in the child’s bedroom). A separate model was used here as heavy condensation was moderately correlated with dampness of the house

Similar associations were observed when the study sample was restricted to those who did not move home between the antenatal and 2 year interviews (number of children = 3258; number of ARI events = 517); when the follow-up period was restricted to the first 2 years (number of children = 6112; number of ARI events = 711); and when the outcome events were restricted to ARIs that had occurred in the winter and spring (number of children = 6112; number of ARI events = 686) (Additional file 1: Table S4). The association with using unflued gas heater remained significant when the study sample was restricted to those who did not move home between the antenatal and 2 year interviews.

In more detailed analysis, the risk of being hospitalised with an ARI was significantly increased if a gas heater was used solely as a form of heating (Table 4).

**Table 4:** Associations between use of gas heaters and hospital admission for an acute respiratory infection during the first five years of life

Type of heating used in the household	Incidence per 1000 person-years (95% CI)	Crude HR (95% CI)	Adjusted HR^a^ (95% CI)
All gas heaters			
Use gas heater only	75.68 (60.70-93.23)	2.44 (1.92-3.10)	1.64 (1.29-2.09)
Use gas heater as well as other forms of heating	23.92 (20.13-28.20)	0.77 (0.63-0.94)	0.82 (0.68-0.99)
Use other forms of heating only	31.04 (28.66-33.57)	1.00	1.00
Unflued gas heaters			
Use unflued gas heater only	74.23 (52.55-101.84)	2.36 (1.66-3.35)	1.48 (1.05-2.09)
Use unflued gas heater as well as other forms of heating	23.73 (18.47-30.03)	0.76 (0.58-0.98)	0.73 (0.57-0.94)
Use other forms of heating only	31.42 (29.21-33.76)	1.00	1.00

^a^Adjusted for all maternal and child factors as mentioned in Table 3 and environmental factors (household tenure, crowding, household smoking, dampness of the house and mould or mildew in the child’s bedroom)

## Discussion

In this ethnically and socioeconomically diverse child cohort, into which participants were enrolled antenatally, the risk of ARI hospitalisation was increased for children living in households that used a gas heater in the room where the child slept at night during their first year of life. The risk was also increased in households who used gas heating as the sole form of heating. The risk was reduced in households that used electric heaters or wood burners as a form of household heating.

The demographic characteristics of our cohort align with all births in NZ over a time period (2007-10) that encompasses study recruitment [22]. Comprehensive information across six interconnected domains was collected, thus allowing a wide range of influences on respiratory health to be considered in multivariate analyses and reducing the risk of residual confounding. In this prospective study, the participants were followed through face-to-face interviews as well as record linkage to routinely collected health data, thereby minimising potential biases associated with loss to follow-up [25] and self-reported data [26].

When interpreting the findings, some limitations should be kept in mind. First, this analysis excluded about 10% of the children enrolled in the study as their mothers did not complete the nine month interviews (*n* = 375) and/or linkage to their hospital discharge data was not established (*n* = 311). The maternal and child characteristics of those not included differed from those included in the analysis. In general, those not included were less socioeconomically advantaged and had poorer health behaviours. This bias is likely to underestimate observed associations between the internal living environment and risk of ARI hospitalisation as the most disadvantaged population in NZ are most likely to have hospital admissions for ARI, and live in poor housing conditions [27]. Second, this study relied on self-report of home environmental factors. As these environmental factors were measured at the interview completed when the cohort children were nine months old, reverse causation (infant illness leading to heightened awareness and reporting of environmental risks) cannot be excluded. However, it is unlikely that the home environment changed considerably from the time of birth until age nine months, and the findings did not change when we restricted our analyses to those who did not move home between the antenatal and 2 year interviews. Third, as the study outcome data were collected through linkage to routinely collected databases, case ascertainment may be affected by inaccuracies in individual data sources [28].

In this cohort, during the first five years, there were 33 ARI episodes per 1000 children-per-year that require hospital inpatient treatment – a higher rate than those reported in a recent global assessment (17 episodes per 1000 children-per-year in the Western Pacific, 20 episodes per 1000 children-per-year in Americas, 7 episodes per 1000 children-per-year in Europe and 10 episodes per 1000 children-per-year in industrialised countries) [29]. An explanation may be the relatively poor condition of the home environment in NZ. Out of 30 OECD (Organisation for Economic Co-operation and Development) countries, NZ ranks 20th and 17th respectively on the proportion of children living in crowded houses or poor households [30].

Consistent with census data [31], almost half of the participants reported living in rented accommodation. Previous studies have treated housing tenure as a marker of socioeconomic status and have linked it to a number of health conditions [32–34]. However, in this study, no independent association of rental accommodation with risk of ARI hospitalisation was evident after controlling for a range of maternal factors including demographics, health status, use of supplements, smoking and pregnancy history.

Household quality is a contemporary issue of concern in NZ. The most recent national survey of housing conditions found one-in-four sampled houses had some degree of dampness, and one-in-five houses had mould evident in at least one room [35]. In this study, one-in-five mothers reported “quite often” or “always or almost always” dampness of the house and heavy condensation in the child’s room, and one-in-eight mothers reported the presence of mould or mildew in the walls or ceilings of the child’s room. Dampness and mould in homes has been linked to a number of respiratory health problems [8, 9]. This analysis showed a dose-response relationship in crude estimates between dampness of the house and ARIs. However, the association was no longer statistically significant after adjustment for maternal and child characteristics. Counterintuitively, after adjustment, we observed a reduced risk of ARI associated with mould or mildew in the child’s room but this may reflect greater awareness of home environmental issues and thus increased focus on maintaining health among parents who reported mould or mildew in the child’s room.

A study on household energy use in NZ reported that at least one third of houses were poorly insulated and generally cold in the winter with the average indoor temperature below the WHO recommended minimum of 18 °C [36]. While solid fuel burners and portable electric heaters are most commonly used to heat the house in NZ, in 2006 about a third of households used unflued gas heaters [36]. Patterns of household heating are changing [37]; and in this study, one-in-seven mothers reported using a flued gas heater and one-in-eight an unflued gas heater for household heating. We found that gas heaters if used to heat the child’s room or used as the only form of household heating increased the risk of ARIs before age 5 years. Gas heaters, especially if unflued, emit moisture and a number of pollutants including nitrogen dioxide, carbon monoxide, carbon dioxide and formaldehyde, thereby affecting respiratory health in children [14, 38]. In a NZ-based trial, replacing unflued gas heaters with non-polluting, more effective heating (heat pumps, enclosed wood burner or flued gas heating) resulted in a significant reduction in both levels of nitrogen dioxide and symptoms of asthma [39, 40].

In NZ, 18% of adults smoke cigarettes regularly [41], and as a result approximately one-third of children are potentially exposed to second hand cigarette smoke [42, 43]. In this study, one-in-seven mothers reported smoking at least one cigarette a day and one-in-three reported smoking by at least one other member of the household. Passive smoking is a well-known risk factor for a number of respiratory illnesses in early childhood [13, 44]. A previous birth cohort study in New Zealand reported an increased risk of ARI associated with maternal smoking in the first two years of life but the association was not significant after two years [45]. In this study, smoking by mother or other members of the household was not associated with the risk of ARI episodes in the first five years of life after adjustment for maternal demographics and other characteristics. Similar results were observed when the analysis was restricted to ARI events in the first two years of life. We conclude that voluntary smoke-free home rules and changes in social norms regarding the acceptability of smoking around non-smokers mean that children, including those from households containing smokers, are less exposed to second hand smoke than in the past [17, 18].

## Conclusions

The burden of ARIs requiring hospital admission is considerable among children involved in this nationally representative cohort and the risk of ARI hospitalisation before age five years is significantly associated with the use of gas heaters in the child’s room in infancy and of gas heaters as the sole form of household heating. These findings underscore the need for more efforts to reduce reliance on gas heaters in NZ households, particularly in the child’s room, alongside other measures to improve housing conditions and the internal living environments for children.

## Additional file

Additional file 1: Supplementary tables. (DOCX 41 kb)

## Abbreviations

ARI: Acute respiratory infection
CAPI: Computer assisted personal interview
CI: Confidence interval
DHB: District Health Board
HR: Hazard ratio
ICD-10-AM: International Statistical Classification of Diseases and Related Health Problems, Tenth Revision, Australian Modification
NZ: New Zealand

## References

[1] Zar HJ, Ferkol TW (2014). The global burden of respiratory disease—Impact on child health. Pediatr Pulmonol.

[2] Craig E, Adams J, Oben G, Reddington A, Wicken A (2013). Simpson J (on behalf of the New Zealand Child and Youth Epidemiology Service). The Health Status of Children and Young People in New Zealand.

[3] Hasegawa K, Tsugawa Y, Brown DF, Mansbach JM, Camargo CA (2013). Trends in bronchiolitis hospitalizations in the United States, 2000-2009. Pediatrics.

[4] Cheung CR, Smith H, Thurland K, Duncan H, Semple MG (2013). Population variation in admission rates and duration of inpatient stay for bronchiolitis in England. Arch Dis Child.

[5] Griffin MR, Zhu Y, Moore MR, Whitney CG, Grijalva CG (2013). U.S. hospitalizations for pneumonia after a decade of pneumococcal vaccination. N Engl J Med.

[6] Grant CC, Scragg R, Tan D, Pati A, Aickin R, Yee RL (1998). Hospitalization for pneumonia in children in Auckland, New Zealand. J Paediatr Child Health.

[7] Grant CC, Wall CR, Gibbons MJ, Morton SM, Santosham M, Black RE (2011). Child nutrition and lower respiratory tract disease burden in New Zealand: A global context for a national perspective. J Paediatr Child Health.

[8] Fisk WJ, Lei-Gomez Q, Mendell MJ (2007). Meta-analyses of the associations of respiratory health effects with dampness and mold in homes. Indoor Air.

[9] Fisk W, Eliseeva E, Mendell M (2010). Association of residential dampness and mold with respiratory tract infections and bronchitis: a meta-analysis. Environ Health.

[10] Colosia AD, Masaquel A, Hall CB, Barrett AM, Mahadevia PJ, Yogev R (2012). Residential crowding and severe respiratory syncytial virus disease among infants and young children: A systematic literature review. BMC Infect Dis.

[11] Baker M, McDonald A, Zhang J, Howden-Chapman P (2013). Infectious diseases attributable to household crowding in New Zealand: A systematic review and burden of disease estimate.

[12] Dherani M, Pope D, Mascarenhas M, Smith KR, Weber M, Bruce N (2008). Indoor air pollution from unprocessed solid fuel use and pneumonia risk in children aged under five years: a systematic review and meta-analysis. Bull World Health Organ.

[13] Jones LL, Hashim A, McKeever T, Cook DG, Britton J, Leonardi-Bee J (2011). Parental and household smoking and the increased risk of bronchitis, bronchiolitis and other lower respiratory infections in infancy: systematic review and meta-analysis. Respir Res.

[14] Fuentes-Leonarte V, Ballester F, Tenías JM. Sources of Indoor Air Pollution and Respiratory Health in Preschool Children. J Environ Public Health 2009;doi: 10.1155/2009/727516. 10.1155/2009/727516 PMC2820286 20168984

[15] Centers for Disease Control and Prevention (2015). Vital Signs: Disparities in nonsmokers’ exposure to secondhand smoke — United States, 1999–2012. Morb Mortal Wkly Rep.

[16] Jarvis MJ, Feyerabend C (2015). Recent trends in children’s exposure to second-hand smoke in England: cotinine evidence from the Health Survey for England. Addiction.

[17] Marsh L, McGee R, Gray A, Newcombe R, Patterson R (2012). Youth experiences of secondhand smoke exposure in New Zealand: evidence from 5 national surveys (2000 to 2008). N Z Med J.

[18] Woodward A (2015). Commentary on Jarvis & Feyerabend (2015): A truly smoke-free upbringing, once rare, is now commonplace. Addiction.

[19] Morton SMB, Atatoa Carr PE, Grant CC, Robinson EM, Bandara DK, Bird A (2013). Cohort profile: growing up in New Zealand. Int J Epidemiol.

[20] Chappell P (2012). The climate and weather of Auckland.

[21] Chappell P (2012). The climate and weather of Waikato.

[22] Morton SMB, Ramke J, Kinloch J, Grant CC, Carr PA, Leeson H (2015). Growing Up in New Zealand cohort alignment with all New Zealand births. Aust N Z J Public Health.

[23] Ministry of Health (2012). National Immunisation Register.

[24] Ministry of Health (2012). National Minimum Dataset (Hospital Inpatient events): Data Mart - Data Dictionary V7.5.

[25] Greenland S (1977). Response and follow-up bias in cohort studies. Am J Epidemiol.

[26] van de Mortel TF (2008). Social desirability response bias in self-report research. Aust J Adv Nurs.

[27] Simpson J, Duncanson M, Oben G, Wicken A, Pierse N (2015). Child Poverty Monitor 2015 Technical Report.

[28] Davie G, Langley J, Samaranayaka A, Wetherspoon ME (2008). Accuracy of injury coding under ICD-10-AM for New Zealand public hospital discharges. Inj Prev.

[29] Nair H, Simões EAF, Rudan I, Gessner BD, Azziz-Baumgartner E, Zhang JSF (2013). Global and regional burden of hospital admissions for severe acute lower respiratory infections in young children in 2010: a systematic analysis. Lancet.

[30] Organisation for Economic Cooperation and Development (OECD) (2009). Doing better for children.

[31] Statistics New Zealand (2014). Housing in Auckland: Trends in housing from the Census of Population and Dwellings 1991 to 2013.

[32] Chaudhuri N (2004). Interventions to improve children’s health by improving the housing environment. Rev Environ Health.

[33] Robertson T, Benzeval M, Whitley E, Popham F (2015). The role of material, psychosocial and behavioral factors in mediating the association between socioeconomic position and allostatic load (measured by cardiovascular, metabolic and inflammatory markers). Brain Behav Immun.

[34] Galobardes B, Shaw M, Lawlor DA, Lynch JW (2006). Indicators of socioeconomic position (part 1). J Epidemiol Community Health.

[35] Buckett NE, Marston NE, Saville-Smith K, Jowett J, Jones M (2011). Preliminary BRANZ House Condition Survey Report. BRANZ Study Report 240.

[36] Isaacs NE, Camilleri M, French L, Pollard A, Saville-Smith K, Fraser R (2006). Energy use in New Zealand Households: Report on the Year 10 Analysis for the Household Energy End-use Project (HEEP). BRANZ Study Report 155.

[37] Statistics New Zealand (2014). 2013 Census QuickStats about housing.

[38] Environmental Health Standing Committee (enHealth) (2007). The health effects of unflued gas heater use in Australia.

[39] Gillespie-Bennett J, Pierse N, Wickens K, Crane J, Nicholls S, Shields D (2008). Sources of nitrogen dioxide (NO2) in New Zealand homes: findings from a community randomized controlled trial of heater substitutions. Indoor Air.

[40] Howden-Chapman P, Pierse N, Nicholls S, Gillespie-Bennett J, Viggers H, Cunningham M (2008). Effects of improved home heating on asthma in community dwelling children: randomised controlled trial. Br Med J.

[41] Ministry of Health (2012). The Health of New Zealand Adults 2011/12: Key findings of the New Zealand Health Survey.

[42] Bowie C (2012). Environmental Health Indicators for New Zealand. Indicator: Child exposure to second-hand smoke in New Zealand households.

[43] Glover M, Hadwen G, Chelimo C, Scragg R, Bullen C, Gentles D (2013). Parent versus child reporting of tobacco smoke exposure at home and in the car. N Z Med J.

[44] Öberg M, Jaakkola MS, Woodward A, Peruga A, Prüss-Ustün A (2011). Worldwide burden of disease from exposure to second-hand smoke: a retrospective analysis of data from 192 countries. Lancet.

[45] Fergusson DM, Hons B, Horwood LJ (1985). Parental smoking and respiratory illness during early childhood: A six-year longitudinal study. Pediatr Pulmonol.

