# Supplementary material for: Internal living environment and respiratory disease in children: findings from the Growing Up in New Zealand longitudinal child cohort study
Source: Environ Health. 2016 Dec 8;15:120. doi: 10.1186/s12940-016-0207-z (PMC5146862; doi:10.1186/s12940-016-0207-z)
Supplement: Additional file 1: — Supplementary tables. (DOCX 41 kb) [file 12940_2016_207_MOESM1_ESM.docx]

#### Table S1. ICD-10 codes used

| **ICD-10 code** | **Disease** |
| --- | --- |
| A15 | Respiratory tuberculosis, bacteriologically and histologically confirmed |
| A16 | Respiratory tuberculosis, not confirmed bacteriologically or histologically |
| A19 | Miliary tuberculosis |
| A36 | Diphtheria |
| A37 | Whooping cough |
| A71 | Trachoma |
| B26 | Mumps |
| B30 | Viral conjunctivitis |
| H00 | Hordeolum and chalazion |
| H01 | Other inflammation of eyelid |
| H04 | Disorders of lacrimal system |
| H10 | Conjunctivitis |
| H60 | Otitis externa |
| H65 | Nonsuppurative otitis media |
| H66 | Suppurative and unspecified otitis media |
| H67 | Otitis media in diseases classified elsewhere |
| H70 | Mastoiditis and related conditions |
| H71 | Cholesteatoma of middle ear |
| H72 | Perforation of tympanic membrane |
| H73 | Other disorders of tympanic membrane |
| H74 | Other disorders of middle ear and mastoid |
| H75 | Other disorders of middle ear and mastoid in diseases classified elsewhere |
| H83 | Other diseases of inner ear |
| J00-J06 | Acute upper respiratory infections |
| J09-J18 | Influenza and pneumonia |
| J20-J22 | Other acute lower respiratory infections |
| J30-J39 | Other diseases of upper respiratory tract |
| J40-J47 | Chronic lower respiratory diseases |
| J86 | Pyothorax |
| J90 | Pleural effusion, not elsewhere classified |
| J91 | Pleural effusion in conditions classified elsewhere |
| J92 | Pleural plaque |
| J93 | Pneumothorax |
| J95 | Postprocedural respiratory disorders, not elsewhere classified |
| J96 | Respiratory failure, not elsewhere classified |
| J98 | Other respiratory disorders |
| J99 | Respiratory disorders in diseases classified elsewhere |

#### Table S2. Characteristics of the mothers of the cohort children included vs. excluded in the analysis

|  |  | **Included (N=6112)** | **Excluded (N=648)** | **p-value** |
| --- | --- | --- | --- | --- |
| **Maternal demographics** |  |  |  |  |
| Age (N=6759) | Mean (SD) | 30.2 (5.9) | 29.0 (6.2) | 0.2 |
| Self-prioritised ethnicity (N=6740) | N (%) |  |  |  |
| European |  | 3338 (55) | 248 (39) | <0.001 |
| Māori |  | 809 (13) | 130 (20) |  |
| Pacific |  | 849 (14) | 137 (21) |  |
| Asian |  | 891 (15) | 101 (16) |  |
| Others |  | 209 (3) | 28 (4) |  |
| Education (N=6740) | N (%) |  |  |  |
| Primary |  | 405 (7) | 74 (12) | <0.001 |
| Secondary |  | 1429 (23) | 179 (28) |  |
| Tertiary |  | 4262 (70) | 391 (61) |  |
| Area of residence (N=6759) | N (%) |  |  |  |
| Main urban |  | 5233 (86) | 541 (84) | 0.09 |
| Other urban |  | 457 (8) | 64 (10) |  |
| Rural |  | 422 (7) | 42 (7) |  |
| Neighbourhood deprivation (NZDep06) (N=6757) | N (%) |  |  |  |
| 1-2 (least deprived) |  | 1001 (16) | 92 (14) | <0.001 |
| 3-4 |  | 1139 (19) | 90 (14) |  |
| 5-6 |  | 1066 (17) | 93 (14) |  |
| 7-8 |  | 1290 (21) | 126 (19) |  |
| 9-10 (most deprived) |  | 1614 (26) | 246 (38) |  |
| **Maternal health** |  |  |  |  |
| Pre-pregnancy BMI (N=5922) | Mean (SD) | 25.4 (6.0) | 25.3 (5.5) | 0.02 |
| Pre-pregnancy self-rated health (N=6745) | N (%) |  |  |  |
| Poor |  | 131 (2) | 19 (3) | 0.02 |
| Fair |  | 477 (8) | 62 (10) |  |
| Good |  | 2057 (34) | 240 (37) |  |
| Very good |  | 2189 (36) | 195 (30) |  |
| Excellent |  | 1246 (20) | 129 (20) |  |
| History of asthma (N=6747) | N (%) |  |  |  |
| Never |  | 4666 (77) | 508 (78) | 0.51 |
| Before pregnancy only |  | 912 (15) | 88 (14) |  |
| Before and during pregnancy or during pregnancy only |  | 522 (8) | 51 (8) |  |
| **Maternal use of supplements** |  |  |  |  |
| Use of folic acid supplements |  |  |  |  |
| In the 3 months before becoming pregnant (N=6114) | N (%) |  |  |  |
| Yes |  | 2201 (40) | 168 (29) | <0.001 |
| No |  | 3327 (60) | 418 (71) |  |
| In the first three months of pregnancy (N=6115) | N (%) |  |  |  |
| Yes |  | 4497 (81) | 386 (66) | <0.001 |
| No |  | 1032 (19) | 200 (34) |  |
| Since the first three months of pregnancy (N=6113) | N (%) |  |  |  |
| Yes |  | 3090 (56) | 283 (48) | 0.002 |
| No |  | 2437 (44) | 303 (52) |  |
| Use of vitamins and minerals |  |  |  |  |
| In the 3 months before becoming pregnant (N=6116) | N (%) |  |  |  |
| Yes |  | 1718 (31) | 128 (22) | <0.001 |
| No |  | 3812 (69) | 458 (78) |  |
| In the first three months of pregnancy (N=6116) | N (%) |  |  |  |
| Yes |  | 2830 (51) | 231 (39) | <0.001 |
| No |  | 2700 (49) | 355 (61) |  |
| Since the first three months of pregnancy (N=6117) | N (%) |  |  |  |
| Yes |  | 2913 (53) | 269 (46) | 0.002 |
| No |  | 2618 (47) | 317 (54) |  |
| **Maternal Smoking** |  |  |  |  |
| Smoked cigarettes during pregnancy (N=6115) | N (%) |  |  |  |
| Yes |  | 568 (10) | 83 (14) | 0.004 |
| No |  | 4962 (90) | 502 (86) |  |
| **Pregnancy history** |  |  |  |  |
| Parity (N=6752) | N (%) |  |  |  |
| First child |  | 2577 (42) | 256 (40) | 0.2 |
| Subsequent child |  | 3528 (58) | 391 (60) |  |
| Pregnancy planning (N=6728) | N (%) |  |  |  |
| Planned |  | 3722 (61) | 339 (52) | <0.001 |
| Unplanned |  | 2360 (39) | 307 (48) |  |

SD – Standard Deviation

#### Table S3. Characteristics of the cohort children included vs. excluded in the analysis

|  |  | **Included (N=6112)** | **Excluded^a^ (N=648)** | **p-value** |
| --- | --- | --- | --- | --- |
| **Child characteristics** |  |  |  |  |
| Gender (N=6760) | N (%) |  |  |  |
| Boy |  | 3165 (52) | 328 (51) | 0.6 |
| Girl |  | 2947 (48) | 320 (49) |  |
| Gestation (N=6743) | N (%) |  |  |  |
| Preterm (<37 weeks) |  | 351 (6) | 29 (5) | 0.2 |
| Term (37-41 weeks) |  | 5608 (92) | 589 (92) |  |
| Postterm (>41 weeks) |  | 145 (2) | 21 (3) |  |
| Birth-weight (g) (N=6750) | Mean (SD) | 3496.8 (571.8) | 3499.1 (531.9) | 0.02 |
| Season of birth (N=6758) | N (%) |  |  |  |
| Summer |  | 1754 (29) | 186 (29) | 0.2 |
| Autumn |  | 1023 (17) | 116 (18) |  |
| Winter |  | 1479 (24) | 133 (21) |  |
| Spring |  | 1856 (30) | 211 (33) |  |
| **Child health at 9 months** |  |  |  |  |
| Proxy-rated health (N=6390) | N (%) |  |  |  |
| Poor |  | 3659 (60) | 172 (62) |  |
| Fair |  | 1704 (28) | 74 (27) |  |
| Good, very good or excellent |  | 748 (13) | 33 (12) |  |
| Health or developmental problems (N=6385) | N (%) |  |  |  |
| Yes |  | 572 (9) | 23 (8) |  |
| No |  | 5534 (91) | 256 (92) |  |
| **Feeding practices** |  |  |  |  |
| Exclusive breastfeeding (N=6310) | N (%) |  |  |  |
| 6+ months |  | 1376 (23) | 78 (29) |  |
| <6 months |  | 4662 (77) | 194 (71) |  |
| Other feedings |  |  |  |  |
| Formula feeding (N=6366) | N (%) |  |  |  |
| Yes |  | 4767 (78) | 203 (73) |  |
| No |  | 1321 (22) | 75 (27) |  |
| Has tried both fruit and vegetable (N=6362) | N (%) |  |  |  |
| Yes |  | 5827 (96) | 267 (97) |  |
| **Time spent outdoors** |  |  |  |  |
| Average hours spent outdoors on a week day (N=6335) | N (%) |  |  |  |
| 1+ hours |  | 4244 (70) | 191 (69) |  |
| <1 hour |  | 1814 (30) | 86 (31) |  |
| Average hours spent outdoors on a weekend day (N=6355) | N (%) |  |  |  |
| 1+ hours |  | 4878 (80) | 233 (84) |  |
| <1 hour |  | 1201 (20) | 43 (16) |  |
| **Child immunisation** |  |  |  |  |
| Received all six vaccination doses on time (N=5802) | N (%) |  |  |  |
| Yes |  | 4695 (86) | 257 (82) |  |
| No |  | 792 (14) | 58 (18) |  |

a Excluded the second or subsequent child from multiple births (N=93).

SD – Standard Deviation

#### Table S4. Sensitivity analyses

| **Variables** | **Adjusted HR^a^** | | |
| --- | --- | --- | --- |
|  | **Analysis 1** | **Analysis 2** | **Analysis 3** |
|  |  |  |  |
| Housing tenure |  |  |  |
| Owner occupancy | 1.00 | 1.00 | 1.00 |
| Tenancy | 0.85 (0.68-1.05) | 0.99 (0.83-1.18) | 1.02 (0.85-1.21) |
| Crowding index |  |  |  |
| <1-2 (low and medium) | 1.00 | 1.00 | 1.00 |
| 2+ (high) | 1.08 (0.85-1.37) | 1.15 (0.95-1.39) | 1.15 (0.94-1.39) |
| Maternal smoking |  |  |  |
| Yes | 1.32 (0.88-1.97) | 1.17 (0.86-1.59) | 1.21 (0.87-1.67) |
| No | 1.00 | 1.00 | 1.00 |
| Smoking by others in the household |  |  |  |
| Yes | 1.02 (0.80-1.30) | 0.90 (0.73-1.11) | 0.89 (0.72-1.10) |
| No | 1.00 | 1.00 | 1.00 |
| Heating the house |  |  |  |
| Yes | 0.77 (0.58-1.04) | 0.82 (0.66-1.02) | 0.83 (0.66-1.05) |
| No | 1.00 | 1.00 | 1.00 |
| Form/s of heating used in the house (N=5298) |  |  |  |
| Electricity |  |  |  |
| Yes | 0.80 (0.61-1.05) | 0.79 (0.63-0.99) | 0.69 (0.55-0.86) |
| No | 1.00 | 1.00 | 1.00 |
| Flued gas heater |  |  |  |
| Yes | 0.80 (0.61-1.05) | 1.14 (0.89-1.45) | 1.21 (0.96-1.54) |
| No | 1.00 | 1.00 | 1.00 |
| Unflued portable gas heater |  |  |  |
| Yes | 0.79 (0.57-1.10) | 0.81 (0.62-1.06) | 0.92 (0.71-1.19) |
| No | 1.00 | 1.00 | 1.00 |
| Wood |  |  |  |
| Yes | 0.81 (0.64-1.02) | 0.72 (0.58-0.88) | 0.79 (0.64-0.97) |
| No | 1.00 | 1.00 | 1.00 |
| Form/s of heating used in the room where child sleeps at night (N=5298) |  |  |  |
| No heating |  |  |  |
| Yes | 0.97 (0.73-1.29) | 0.73 (0.57-0.93) | 0.81 (0.64-1.04) |
| No | 1.00 | 1.00 | 1.00 |
| Electricity |  |  |  |
| Yes | 0.89 (0.71-1.13) | 1.05 (0.86-1.28) | 0.89 (0.73-1.08) |
| No | 1.00 | 1.00 | 1.00 |
| Flued gas heater |  |  |  |
| Yes | 0.95 (0.55-1.66) | 1.92 (1.28-2.88) | 2.08 (1.41-3.07) |
| No | 1.00 | 1.00 | 1.00 |
| Unflued portable gas heater |  |  |  |
| Yes | 2.04 (1.10-3.77) | 1.66 (1.03-2.69) | 2.07 (1.30-3.30) |
| No | 1.00 | 1.00 | 1.00 |
| Wood |  |  |  |
| Yes | 1.30 (0.84-2.02) | 0.87 (0.57-1.33) | 1.13 (0.77-1.65) |
| No | 1.00 | 1.00 | 1.00 |
| Dampness of the house |  |  |  |
| Never of hardly ever | 1.00 | 1.00 | 1.00 |
| Not very often | 1.09 (0.87-1.35) | 0.96 (0.79-1.15) | 1.02 (0.85-1.23) |
| Quite often | 1.28 (0.99-1.66) | 1.08 (0.86-1.34) | 1.11 (0.89-1.39) |
| Always or almost always | 1.22 (0.81-1.82) | 1.18 (0.87-1.59) | 1.12 (0.82-1.55) |
| Heavy condensation in the room where child sleeps at night |  |  |  |
| Never of hardly ever | 1.00 | 1.00 | 1.00 |
| Not very often | 1.06 (0.85-1.31) | 1.02 (0.84-1.23) | 1.04 (0.86-1.25) |
| Quite often | 1.16 (0.89-1.50) | 1.15 (0.92-1.42) | 1.05 (0.84-1.31) |
| Always or almost always | 0.86 (0.58-1.29) | 1.08 (0.80-1.47) | 0.95 (0.69-1.31) |
| Mould or mildew in the walls or ceilings in the room where child sleeps at night in the past two weeks |  |  |  |
| Yes | 0.58 (0.42-0.,79) | 0.93 (0.74-1.16) | 0.87 (0.69-1.10) |
| No | 1.00 | 1.00 | 1.00 |

a Adjusted for all maternal factors, child factors and environmental factors as mentioned in Table 3.

Analysis 1 – restricted the study sample to those who did not move home between the antenatal and 2 year interviews (Number of children=3258; Number of ARI events=517)

Analysis 2 – restricted the follow-up period to age 2 years (Number of children=6112; Number of ARI events=711)

Analysis 3 – restricted the outcome events to acute respiratory infections that had occurred in the winter and spring (June-November) (Number of children=6112; Number of ARI events=686)
